# Evaluating deep learning and radiologist performance in volumetric prostate cancer analysis with biparametric MRI and histopathologically mapped slides

**DOI:** 10.1007/s00261-024-04734-6

**Published:** 2024-12-11

**Authors:** Enis C. Yilmaz, Stephanie A. Harmon, Rosina T. Lis, Omer Tarik Esengur, David G. Gelikman, Marcial Garmendia-Cedillos, Maria J. Merino, Bradford J. Wood, Krishnan Patel, Deborah E. Citrin, Sandeep Gurram, Peter L. Choyke, Peter A. Pinto, Baris Turkbey

**Affiliations:** 1 Molecular Imaging Branch, National Cancer Institute, National Institutes of Health, Bethesda, Maryland USA; 2 Instrument Development and Engineering Application Solutions, National Institute of Biomedical Imaging and Bioengineering, National Institutes of Health, Bethesda, Maryland USA; 3 Laboratory of Pathology, National Cancer Institute, National Institutes of Health, Bethesda, Maryland USA; 4 Center for Interventional Oncology, National Cancer Institute, National Institutes of Health, Bethesda, Maryland USA; 5 Department of Radiology, Clinical Center, National Institutes of Health, Bethesda, Maryland USA; 6 Radiation Oncology Branch, National Cancer Institute, National Institutes of Health, Bethesda, Maryland USA; 7 Urologic Oncology Branch, National Cancer Institute, National Institutes of Health, Bethesda, Maryland USA

## Introduction

In the United States, prostate cancer (PCa) remains the most prevalent non-cutaneous malignancy among biologically male individuals and a significant cause of cancer-related mortality (1). The advent and integration of multiparametric MRI (mpMRI) into the diagnostic and management pathways of PCa have marked a pivotal shift in how the disease is approached. MRI not only facilitates the selection of patients for biopsy by identifying likely cancerous lesions (2) but also guides biopsy needles with high precision (3), thereby enhancing the accuracy of tissue diagnoses. Additionally, MRI plays a crucial role in disease monitoring during active surveillance, treatment decision making, and post-therapy assessment, making it an indispensable tool in the continuum of prostate cancer care (4–7).

Accurate determination of tumor extent through MRI is vital particularly in selecting appropriate candidates for focal therapy, to deliver treatment only to the tumorigenic region and spare the rest of the prostate (8). This approach hinges on precise tumor localization and volume estimation, which can significantly impact treatment outcomes and quality of life (9–12). However, challenges persist in the accurate estimation of tumor volume through MRI, particularly with biparametric MRI (bpMRI), which tends to underestimate tumor size (13), notably in tumors with smaller dimensions (14) and lower Gleason scores (15). Dynamic contrast-enhanced (DCE) sequences, while showing the best performance among MRI techniques (13), still exhibit significant discrepancies when compared with whole mount histopathology (WMH), the gold standard in tumor volume determination (16).

This study focuses on leveraging artificial intelligence (AI) to enhance the predictive accuracy of bpMRI, aiming to align its performance more closely with that of WMH-based tumor volume estimations. By comparing AI-enhanced bpMRI interpretations with traditional radiologist contours, this research seeks to underscore potential improvements in treatment planning and patient outcomes.

## Materials and methods

### Study sample

**Fig. 1:**
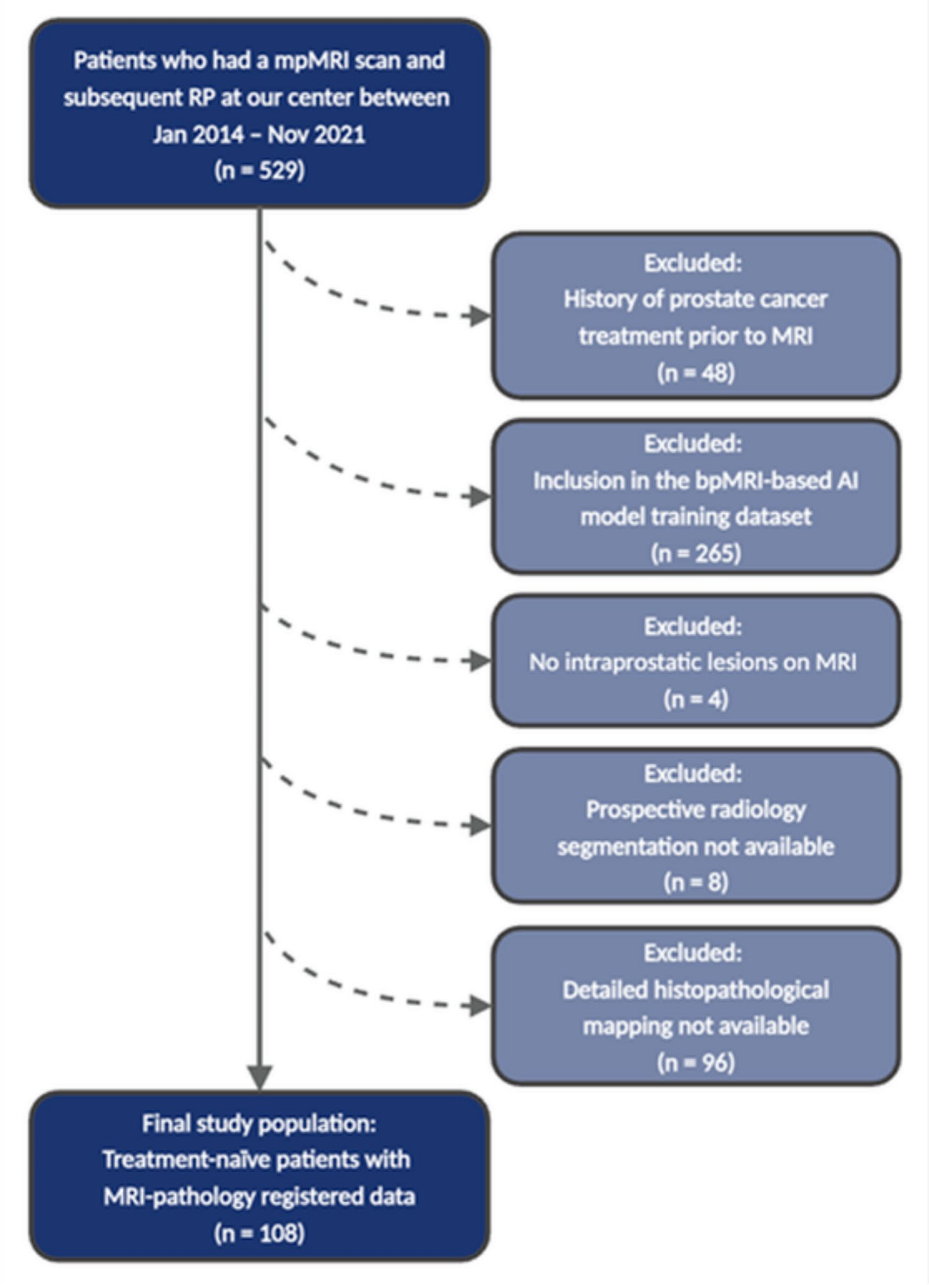
Study flowchart. *Note* bpMRI: Biparametric MRI, mpMRI: Multiparametric MRI, PI-RADS: Prostate Imaging Reporting and Data System, RP: Radical prostatectomy

This HIPAA-compliant retrospective study was conducted with the Institutional Review Board approval. Each patient included in the study provided a written informed consent and were enrolled in at least one of the following protocols: NCT00102544, NCT02594202, NCT03354416. All patients who received mpMRI scan, and subsequently had radical prostatectomy (RP) at our institution between January 2014 to November 2021 were considered for inclusion. Patients who had received other PCa treatments before the MRI, those included in the AI training dataset, those without intraprostatic lesions on MRI, those lacking prospective lesion segmentations, and those without detailed histopathological mapping of the whole-mount specimens were excluded from the study. The final study sample consisted of treatment-naïve patients with MRI-pathology registered data (Fig. 1).

### mpMRI acquisition and interpretation

All patients underwent imaging on a 3T MRI (Achieva 3.0T TX scanner, Philips, Best, The Netherlands or Ingenia Elition 3.0T X, Philips, Best, The Netherlands or Verio 3 T scanner, Siemens, Erlangen, Germany) utilizing phase arrayed surface coil, with or without an endorectal coil. Image acquisitions adhered to PI-RADS guidelines (17, 18) and included the following sequences: T2-weighted imaging (T2WI), high *b*-value diffusion-weighted imaging (DWI), and dynamic contrast-enhanced (DCE) MRI. MRI acquisition parameters are provided in Supplemental Table 1.

One genitourinary radiologist (B.T., with over 15 years of experience in prostate cancer imaging) prospectively assessed each mpMRI according to the PI-RADS v2.0 (from May 2015 to March 2019) or v2.1 (from April 2019 to March 2023). Small number of patients who were scanned prior to the launch of PI-RADS v2.0 were retrospectively evaluated. Planimetric whole prostate organ and intraprostatic lesion segmentations were performed prospectively using a commercial segmentation tool (DynaCAD, Invivo), and lesion maximum diameters were measured on axial images by the same genitourinary radiologist.

### Radical prostatectomy and histopathological annotation

**Fig. 2:**
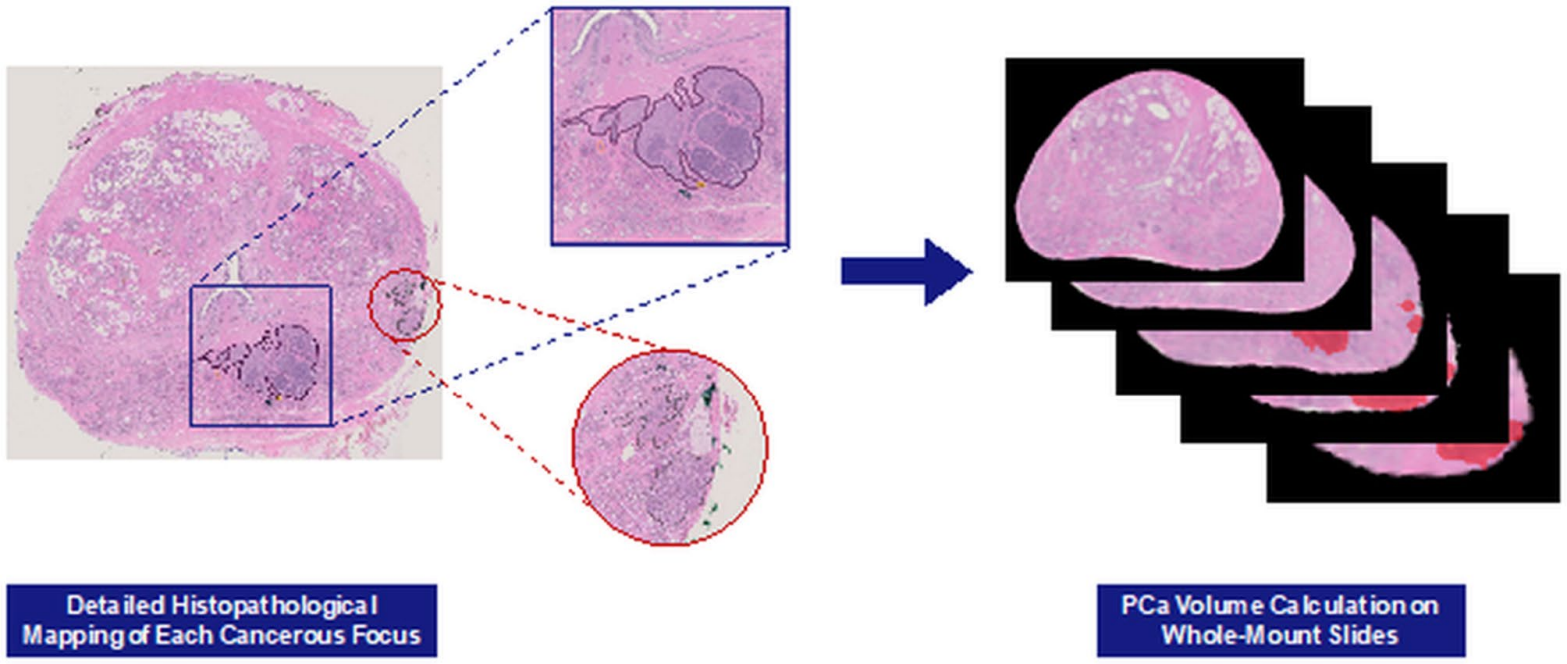
Histopathological mapping and tumor volume calculation on whole-mount slides

Radical prostatectomies were performed by one of the two urologists (P.A.P. > 15 years of experience; S.G > 5 years of experience in prostate surgery) using a robotic approach. Prostate segmentations conducted by the genitourinary radiologist were used to create patient-specific 3D-printed molds as previously described (19, 20). After robotic radical prostatectomy, the specimen was fixed in formalin for 2–24 h, placed in the customized mold, and sliced into 6 mm axial sections. These molds preserve the in-vivo shape of the prostate, facilitating accurate imaging-pathologic correlation and multimodal data analysis (21). Whole-mount sections were then digitally scanned on either an Aperio (obtained at 0.5404 μm/pixel) or Hamamatsu NanoZoomer (obtained at 0.2212 μm/pixel). A genitourinary pathologist (R.T.L., with over 15 years of experience in genitourinary pathology) digitally delineated all tumors on digitized whole-mount slides and categorized each into the appropriate International Society of Urologic Pathology (ISUP) grade group (GG) (Fig. 2) using QuPath software (22). WMH-based tumor volumes were determined from the histopathological annotations, incorporating a shrinkage correction factor of 1.15 (23, 24).

### bpMRI-based AI model and alternative thresholds

All MRIs were evaluated using a previously developed, cascaded, deep-learning-based AI model, available at https://github.com/Project-MONAI/research-contributions/tree/main/prostate-mri-lesion-seg (25). This AI model was trained and validated on a dataset of 1240 patients, comprising both institutional dataset and the publicly available PROSTATEx dataset. An independent test of 150 patients from these datasets were used in the model development study, totaling 1390 patients overall. Training was conducted over a total of 1000 epochs, using the Adam optimizer for both regular model optimization and saliency map refinement, with optimization guided by the soft Dice loss function. Expert lesion segmentations were used as ground truth during algorithm training, with the goal of detecting all suspicious lesions ranging from PI-RADS 2 to 5, simulating a radiologist’s readout. A 3D U-Net-based deep neural network was utilized for lesion detection and segmentation, followed by a 3D residual neural network to filter out benign prostatic hyperplasia nodules. Thresholds in creating binary outputs from probability maps refer to the cutoff values used to classify a region as positive or negative based on its predicted probability. The binary lesion prediction outputs were generated using flexible (50%), default (63%), and strict (75%) probability thresholds of the AI model. The 63% threshold serves as the default and a central point, balancing sensitivity and specificity. The 50% threshold, commonly used in binary classification, is useful in scenarios where detecting all potential lesions is critical, even if some false positives occur. In contrast, the 75% threshold prioritizes specificity, reducing false positives, which can help minimize unnecessary interventions in clinical practice. This range provides practical insight without extending to extreme values, which may be less informative for typical clinical or diagnostic applications.

### Registration of MRI to pathology

To achieve precise registration of digitized whole-mount slides to T2W-MRIs, ProsRegNet, a deep learning model trained on 99 patients and validated with data from 53 patients across three different cohorts, was employed (code available at https://github.com/pimed//ProsRegNet) (26). These cohorts included MRIs with or without an endorectal coil, and histopathology images as whole mounts, quadrants, or at low resolution to enable ProsRegNet to manage variations in imaging modalities and resolution. First, both T2W-MRIs and digitized whole mounts were preprocessed for approximate alignment, accounting for flip/rotation of digital pathology images compared to orientation of the prostate on MRI. Prostate organ boundaries on both MRI and digital pathology images were used to limit the registration area. ProsRegNet then estimated affine and deformable transformations using deep neural networks of intraprostatic features, allowing the accurate mapping of cancer labels from histopathology images onto the corresponding MRI scans (Supplemental Table 2). The final result is a binary mask of cancerous lesions in the spatial orientation and resolution of the MRI. As sections from the radical prostatectomy specimen were taken every 6 mm, interpolation was used to match the output binary mask with the MRI resolution.

### Statistical analysis

Overall, the distribution of radiologist-defined and the AI model-defined (flexible, default, and strict) whole-gland tumor volumes were compared against the gold standard, WMH and to each other using the Wilcoxon signed-rank test. The association between clinical features (PSA, PSA-density), MRI features (PI-RADS, prostate volume), postsurgical pathological features (prostate weight, ISUP GG and extraprostatic extension [EPE] status on WMH) as well as radiologist-, AI-, and WMH-defined tumor volumes was measured with Kendall’s τ coefficient. Bland-Altman plots were generated to evaluate the pattern of relative differences among the volumes. Sub-analyses were performed on groups stratified by presurgical (PSA [< 4, ≥ 4 to < 10, and ≥ 20 ng/mL], PI-RADS scores PI-RADS ≤ 3, PI-RADS 4, and PI-RADS 5], MRI prostate volume[< 34mL, 34-45mL, 45-58mL, and ≥ 58mL]) and postsurgical pathologic features (ISUP GG [GG1, GG2, GG3, and ≥ GG4] and the presence or absence of EPE on WMH). All statistical analyses were conducted using R (version 4.3.3), with *P* values less than 0.05 considered statistically significant. Given the exploratory nature of this study, *P* values were not adjusted for multiplicity.

## Results

### Patient population

A total of 529 patients underwent mpMRI and subsequent RP between January 2014 and November 2021. Exclusions were applied for patients treated prior to MRI (*n* = 48), included in the AI model training dataset (*n* = 265), lacking visible intraprostatic lesions on MRI (*n* = 4), without prospective radiology segmentations (*n* = 8), and without detailed histopathological mapping (*n* = 96). This resulted in a final study population of 108 patients with a median age of 63 years (IQR, 57–68 years) and a median serum prostate-specific antigen level of 7.3 ng/mL (IQR, 5.4–10.7 ng/mL). Regarding family history of prostate cancer, 44% (48/108) were positive, while 3% (3/108) had an unknown family history. The median interval between MRI and RP was 108 days (IQR, 67–157 days) (Table 1).

**Table 1:** Patient demographics and characteristics

Parameter	Unit / Category	All (*n* = 108)
Age	Years	63* [57–68]
Race	African American	27 (25)
	Asian	5 (5)
	Caucasian	71 (66)
	Hawaiian/Pac. Island	1 (1)
	Mixed	2 (2)
	Unknown	2 (2)
Family history	Negative	57 (53)
	Positive	48 (44)
	Unknown	3 (3)
MRI-RP interval	days	108* [67–157]
PSA	ng/mL	7.3* [5.4–10.7]
Prostate volume	cm^3^	45* [34–58]
PSA-density	ng/mL^2^	0.16* [0.10–0.24]
PROSTATE WEIGHT	g	52.3 [44.2–67.2]
Index Lesion– PI-RADS	2	3 (3)
	3	7 (6)
	4	39 (36)
	5	59 (55)
ISUP GG ON WMH	GG 1	4 (4)
	GG 2	60 (56)
	GG 3	25 (23)
	GG 4	14 (13)
	GG 5	5 (5)

*Note* Data are number of patients unless otherwise specified, numbers in parentheses are percentages

ISUP: International Society of Urological Pathology, GG: ISUP grade group, PI-RADS: Prostate Imaging Reporting and Data System, PSA: Prostate-specific antigen, RP: Radical prostatectomy

*Data are median values with IQRs in brackets

### Distribution of PI-RADS scores and ISUP GGs

The distribution of index lesion PI-RADS scores was as follows: 3% (3/108) for PI-RADS 2, 6% (7/108) for PI-RADS 3, 36% (39/108) for PI-RADS 4, and 55% (59/108) for PI-RADS 5. Histopathological review revealed ISUP GG scores of 4% (4/108) for GG 1, 56% (60/108) for GG 2, 23% (25/108) for GG 3, 13% (14/108) for GG 4, and 5% (5/108) for GG 5. The median measured prostate weight on histopathological examination was 52.3 g (IQR, 44.2–67.2 g).

### Comparison of PCa volumes: WMH, radiologist and AI estimations

**Fig. 3:**
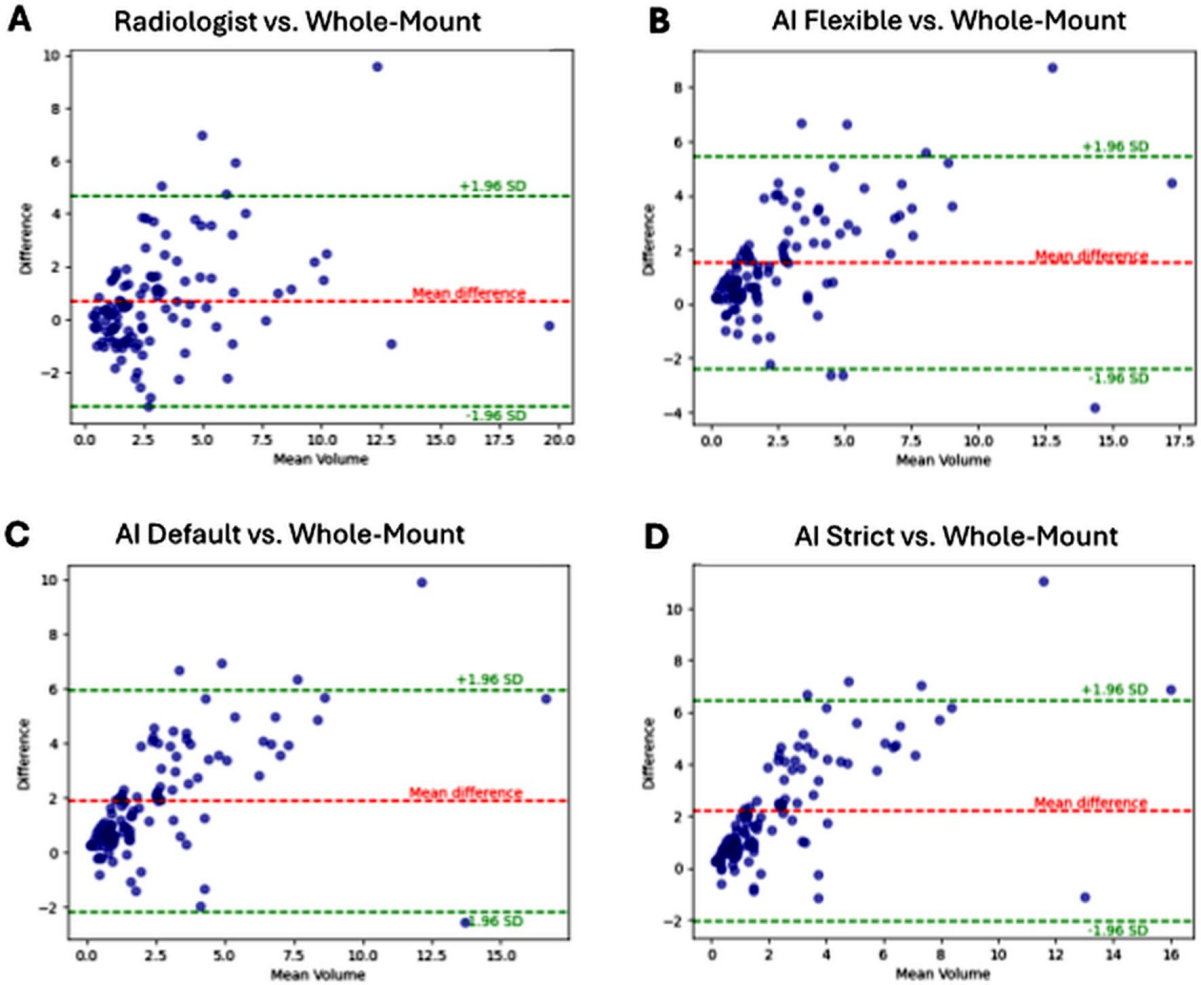
Bland-Altman plots of prostate cancer volumes on whole-mount specimens in comparison to the radiologist contours (**A**) and the bpMRI-based AI algorithm at flexible (**B**), default (**C**), strict (**D**) cutoffs

Out of 108 patients, all radiologist segmentations overlapped with at least one of the WMH-based cancer labels. Contrastingly, the AI failed to identify cancer in 3, 7, and 9 patients at flexible, default, and strict thresholds, respectively. The median PCa volume on WMH was 2.3 mL (IQR, 1.2–4.9 mL), which was higher compared to the volumes delineated by the radiologist (2.1 mL [IQR, 1.2–3.6 mL], *P* =.003) and the AI-based estimations at flexible (1.2 mL [IQR, 0.5–2.7 mL], *P* <.001), default (0.9 mL [IQR, 0.3-2.0 mL], *P* <.001), and strict (0.7 mL [IQR, 0.2–1.8 mL], *P* <.001) thresholds (Table 2). Additionally, the distribution of radiologist-segmented volumes (median of 2.1 mL) was significantly higher than the volumes estimated by the AI at flexible (1.2 mL, *P* <.001), default (0.9 mL, *P* <.001), and strict (0.7 mL, *P* <.001) thresholds. As shown via Bland-Altman plots (Fig. 3), the lowest bias in volume measurement was observed for radiologist-defined volumes as compared to all AI-estimated tumor volumes when judged against WMH as the referent.

**Table 2:** Comparison of prostate cancer volumes (mL) as measured by whole mount histopathology, radiologist, and AI-based estimations (flexible, default, strict thresholds) stratified by index lesion PI-RADS scores (2&3, 4, 5)

Groups and comparisons	Median Volume (mL)	IQR	Median Difference (mL)	*P* value
Overall population (*n* = 108)				
WMH-based volume	2.3	1.2–4.9	Ref	Ref
Radiologist	2.1	1.2–3.6	-0.4	0.003
AI (flexible)	1.2	0.5–2.7	-1.1	< 0.001
AI (default)	0.9	0.3-2.0	-1.3	< 0.001
AI (strict)	0.7	0.2–1.8	-1.6	< 0.001
PI-RADS 5 (*n* = 59)				
WMH-based volume	3.7	1.8–6.7	Ref	Ref
Radiologist	3.1	2.2–4.6	-0.4	0.02
AI (flexible)	1.9	1.1–3.6	-1.7	< 0.001
AI (default)	1.5	0.9-3.0	-2.1	< 0.001
AI (strict)	1.2	0.7–2.5	-2.4	< 0.001
PI-RADS 4 (*n* = 39)				
WMH-based volume	1.3	0.8–2.8	Ref	Ref
Radiologist	1.3	0.8–1.9	-0.2	0.20
AI (flexible)	0.5	0.3–1.1	-0.7	< 0.001
AI (default)	0.4	0.2–0.8	-0.8	< 0.001
AI (strict)	0.3	0.1–0.7	-0.9	< 0.001
PI-RADS 2&3 (*n* = 10)				
WMH-based volume	1.2	0.6–4.1	Ref	Ref
Radiologist	0.7	0.5–1.3	-0.5	0.08
AI (flexible)	0.5	0.1–2.2	-0.8	0.006
AI (default)	0.3	0.0-1.2	-1.0	0.004
AI (strict)	0.2	0.0-0.6	-1.1	0.002

AI: Artificial intelligence, PI-RADS: Prostate Imaging Reporting and Data System, WMH: Whole-mount histopathology

### Subgroup analysis by presurgical features (PSA, PI-RADS categories, prostate volume)

**Fig. 4:**
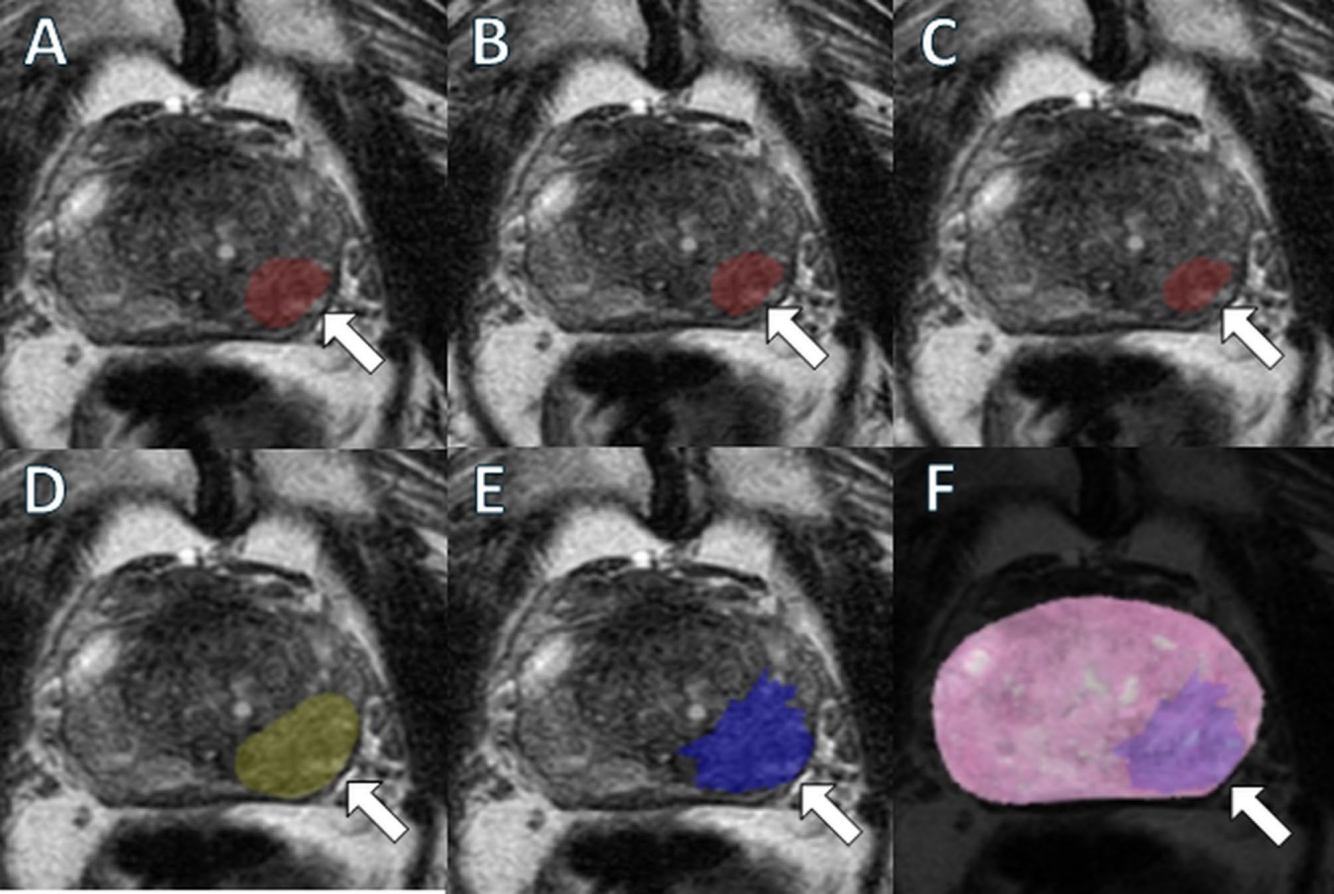
A 77-year-old patient with a serum prostate-specific antigen level of 17.8 ng/mL and a PI-RADS 5 lesion located in the left mid-base peripheral zone. This lesion was delineated using a biparametric MRI-based AI model at flexible (**A**), default (**B**), and strict (**C**) thresholds resulting in annotation volumes of 2.2 mL, 1.5 mL, and 1 mL, respectively, as well as by the radiologist (**D**) with an annotation volume of 4 mL (arrows). Corresponding pathologist annotations of the neoplastic focus are illustrated on T2-weighted imaging (**E**) alongside the related whole-mount histopathology slide (**F**) (arrows) which indicate a volume of 5.7 mL. Histopathological evaluation of the specimen revealed Grade Group 3 prostate adenocarcinoma. *Note* bpMRI: Biparametric MRI, mpMRI: Multiparametric MRI, PI-RADS: Prostate Imaging Reporting and Data System, RP: Radical prostatectomy

For PSA levels < 4 ng/mL (*n* = 11), the median PCa volumes measured by WMH were higher than those estimated by AI for all threshold probabilities (all *P* <.01), but no significant difference was observed between WMH and radiologist measurements (*P* =.64) (Supplemental Table 3). For PSA levels between 4 and 10 ng/mL (*n* = 67), the distribution of WMH volumes was higher as compared to AI-defined volumes (all *P* <.001) but was similar radiologist-defined volumes (*P* =.27). In the 10 to 20 ng/mL PSA group (*n* = 24), both AI- and radiologist-defined volumes underestimated the WMH tumor volume (all *P* <.001) (Fig. 4). In the PSA ≥ 20 ng/mL group, which included only 6 patients, the distribution of volumes obtained by each of the 4 methods under study was similar to the referent, WMH.

**Fig. 5:**
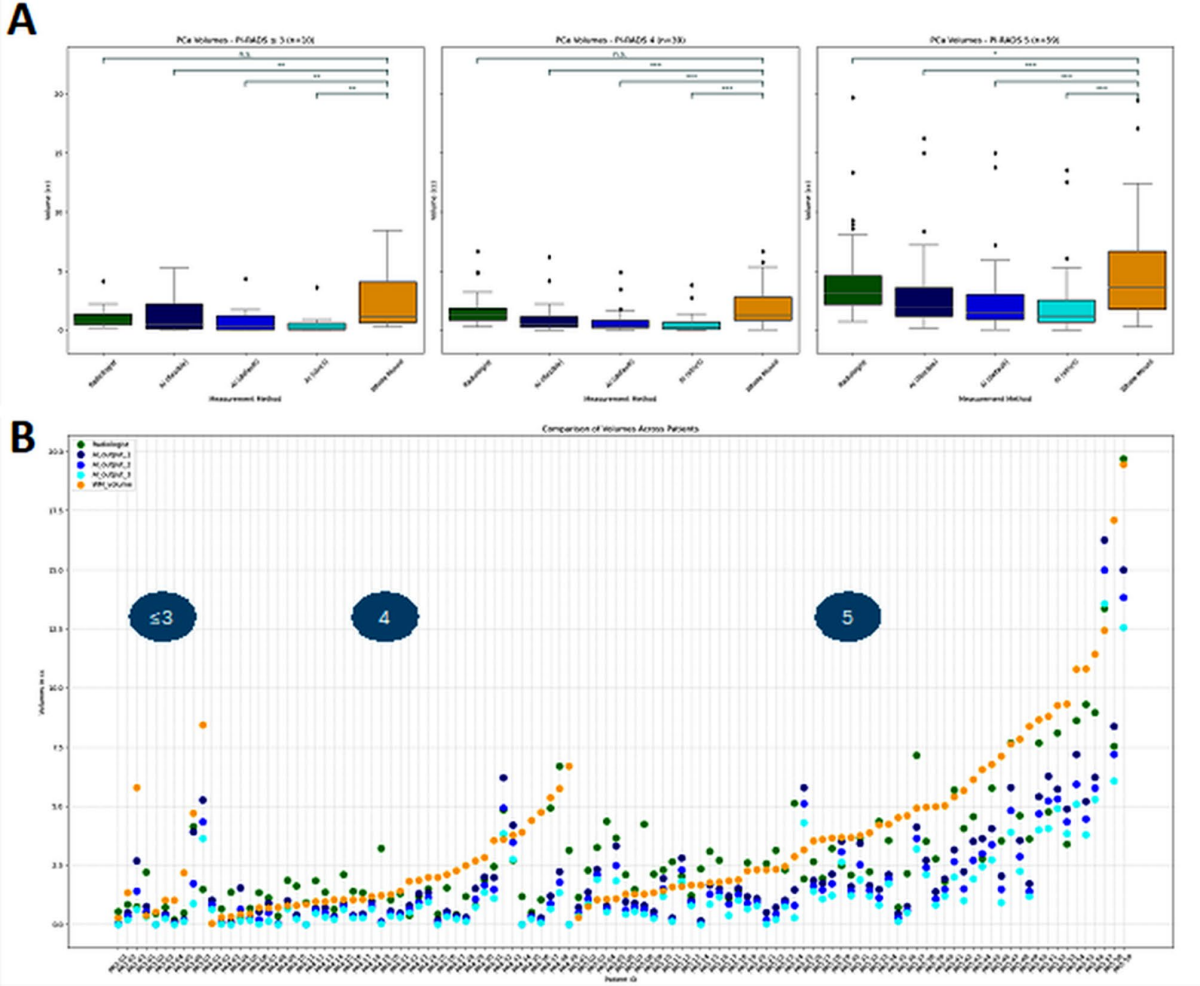
(**A**) Box plots of volumes based on radiologist-based annotations and AI estimations at different thresholds in comparison to the whole-mount based prostate cancer (PCa) volume stratified by the index lesion PI-RADS score. *** indicates *P* ≤.001, ** indicates *P* ≤.01, * indicates *P* ≤.05 and n.s. indicates not significant. (**B**) Demonstration of radiologist and AI estimations of PCa volume (dark blue = flexible, blue = default, teal = strict) on whole-mount histopathology for all patients stratified by index lesion PI-RADS scores. AI: Artificial intelligence, PI-RADS: Prostate Imaging Reporting and Data System, WM: Whole-mount

For patients with PI-RADS 5 index lesion (*n* = 59), WMH volumes (median: 3.7 mL) were underestimated by AI estimations at flexible (median: 1.9 mL), default (median: 1.5 mL) and strict (median: 1.2 mL) thresholds (all *P* <.001) and radiologist measurements (median: 3.1 mL, *P* =.02) (Table 2). For PI-RADS 4 group (*n* = 39), WMH volumes (median: 1.3 mL) were higher compared to AI estimations at all thresholds (all *P* <.001) but were similar to that of the radiologist measurements (median of 1.3 mL, *P* =.20). Within the PI-RADS ≤ 3 group (*n* = 10), the median PCa volumes measured by WMH (median of 1.2 mL) were higher compared to AI estimations at all thresholds (all *P* <.01), with no significant differences observed between WMH and radiologist measurements (*P* =.08) (Fig. 5).

For all gland volume categories, the AI underestimated WMH-based PCa volumes (all, *P* ≤.01). Although the radiologist-defined volumes were similar to WMH measurements in < 34 mL (*n* = 25), 34–45 mL (*n* = 29), and 45–58 mL (*n* = 24) subgroups, these volumes (median: 2.3 mL [IQR, 1.1–3.4 mL]) underestimated the WMH-based PCa volumes (median 3.5 mL [IQR, 1.5–5.7 mL]) (*P* =.04) in the ≥ 58 mL group (*n* = 30) (Table 3).

**Table 3:** Comparison of prostate cancer volumes (mL) as measured by whole mount histopathology, radiologist, and AI-based estimations (flexible, default, strict thresholds) stratified by the gland volume

Groups and comparisons	Median Volume (mL)	IQR	Median Difference (mL)	*P* value
Gland Volume < 34 mL (*n* = 25)				
WMH-based volume	2.2	1.3–4.9	Ref	Ref
Radiologist	2.1	1.2–3.5	-0.4	0.29
AI (flexible)	1.3	0.8–2.7	-0.9	< 0.001
AI (default)	1	0.6–2.3	-1.3	< 0.001
AI (strict)	0.8	0.4–1.9	-1.5	< 0.001
34 ≤ Gland Volume < 45 mL (*n* = 29)				
WMH-based volume	2.8	1.2–6.1	Ref	Ref
Radiologist	2.6	1.9–4.3	-0.6	0.08
AI (flexible)	0.9	0.5–3.5	-1.8	< 0.001
AI (default)	0.8	0.3–2.5	-2.1	< 0.001
AI (strict)	0.6	0.2–1.8	-2.3	< 0.001
45 ≤ Gland Volume < 58 mL (*n* = 24)				
WMH-based volume	1.8	0.8–3.3	Ref	Ref
Radiologist	1.4	1-2.5	-0.2	0.36
AI (flexible)	1	0.4–1.6	-0.6	0.01
AI (default)	0.8	0.3–1.4	-0.9	< 0.001
AI (strict)	0.7	0.2–1.1	-1	< 0.001
Gland Volume ≥ 58 mL (*n* = 30)				
WMH-based volume	3.5	1.5–5.7	Ref	Ref
Radiologist	2.3	1.1–3.4	-1.2	0.04
AI (flexible)	1.6	0.7–2.6	-1.6	< 0.001
AI (default)	1.1	0.5–1.7	-1.8	< 0.001
AI (strict)	0.7	0.2–1.4	-1.9	< 0.001

AI: Artificial intelligence, GG: ISUP grade group, WMH: Whole-mount histopathology

### Subgroup analysis by postsurgical pathology (ISUP GG and EPE status)

For the GG 1 (*n* = 4) subgroup, the distribution of radiologist-defined measurements (*P* =.38) or AI estimations at all thresholds (all, *P* >.05) were similar to the WMH referent. For GG 2 (*n* = 60), WMH volumes were higher compared to AI estimations at all thresholds (all *P* <.001), but no significant differences were found between WMH and radiologist measurements (*P* =.06). For GG 3 (*n* = 25), WMH volumes were higher compared to AI estimations at all thresholds (all *P* <.001), with no significant differences observed between WMH and radiologist measurements (*P* =.08). In the GG 4 & 5 group (*n* = 19), WMH volumes were underestimated by the AI estimations at all thresholds (all *P* <.001) and the radiologist measurements (*P* =.049) (Table 4).

**Table 4:** Comparison of prostate cancer volumes (mL) as measured by whole mount histopathology, radiologist, and AI-based estimations (flexible, default, strict thresholds) across different ISUP grade groups (GG 1, GG 2, GG 3, GG 4 & 5)

Groups and comparisons	Median Volume (mL)	IQR	Median Difference (mL)	*P* value
GG 1 (*n* = 4)				
WMH-based volume	0.4	0.3–0.8	Ref	Ref
Radiologist	1.3	0.9–1.7	0.6	0.38
AI (flexible)	0.9	0.6–1.1	0.1	0.88
AI (default)	0.7	0.5–0.9	0	0.88
AI (strict)	0.5	0.3–0.7	-0.1	0.63
GG 2 (*n* = 60)				
WMH-based volume	1.9	1.2–4.4	Ref	Ref
Radiologist	1.9	1.1–2.9	-0.3	0.06
AI (flexible)	0.9	0.4–1.7	-0.9	< 0.001
AI (default)	0.7	0.3–1.4	-1.2	< 0.001
AI (strict)	0.5	0.2–1.2	-1.5	< 0.001
GG 3 (*n* = 25)				
WMH-based volume	2.3	1.1–3.9	Ref	Ref
Radiologist	2.5	1.2–3.5	-0.4	0.08
AI (flexible)	1.5	0.5–2.3	-0.8	0.004
AI (default)	0.8	0.3–1.7	-0.9	< 0.001
AI (strict)	0.7	0.2–1.2	-1	< 0.001
GG 4 & GG 5 (*n* = 19)				
WMH-based volume	5	3.5–10.0	Ref	Ref
Radiologist	4.6	2.4–7.9	-1.1	0.049
AI (flexible)	3.1	1.8–5.7	-1.9	< 0.001
AI (default)	2.6	1.5–5.1	-2.3	< 0.001
AI (strict)	2	1.1–4.4	-2.5	< 0.001

AI: Artificial intelligence, GG: ISUP grade group, WMH: Whole-mount histopathology

On histopathological examination, 27% (29 out of 108) of the patients were found to have extraprostatic extension. In patients with no evidence of EPE (*n* = 79), the radiologist PCa volume estimation (median 1.8 mL [IQR, 1.1–2.8 mL]) was similar to the PCa volume on WMH (median 1.8 mL [IQR, 1.0-3.8 mL]) (*P* =.16). However, in patients with EPE on WMH (*n* = 29), the radiologist measurement (median 3.4 mL [IQR, 2.3–5.8 mL]) was lower than the WMH-based PCa volume (median 4.4 mL [IQR, 2.3–6.8 mL]) (*P* =.002). AI estimations at flexible, default and strict thresholds underestimated the PCa volume, irrespective of the presence of EPE (Table 5).

**Table 5:** Comparison of prostate cancer volumes (mL) as measured by whole mount histopathology, radiologist, and AI-based estimations (flexible, default, strict thresholds) stratified by the presence of extraprostatic extension on whole-mount histopathology

Groups and comparisons	Median Volume (mL)	IQR	Median Difference (mL)	*P* value
EPE negative (*n* = 79)				
WMH-based volume	1.9	1.0-3.8	Ref	Ref
Radiologist	1.8	1.1–2.8	-0.1	0.16
AI (flexible)	1	0.5-2.0	-0.7	< 0.001
AI (default)	0.8	0.3–1.5	-0.9	< 0.001
AI (strict)	0.6	0.2–1.2	-1	< 0.001
EPE positive (*n* = 29)				
WMH-based volume	4.4	2.3–6.8	Ref	Ref
Radiologist	3.4	2.3–5.8	-1.1	0.002
AI (flexible)	2	0.7–4.1	-2.2	< 0.001
AI (default)	1.5	0.4–3.6	-2.4	< 0.001
AI (strict)	1.2	0.3–3.2	-2.7	< 0.001

AI: Artificial intelligence, GG: ISUP grade group, WMH: Whole-mount histopathology

### Correlation of clinical, imaging, and histopathologic features

**Fig. 6:**
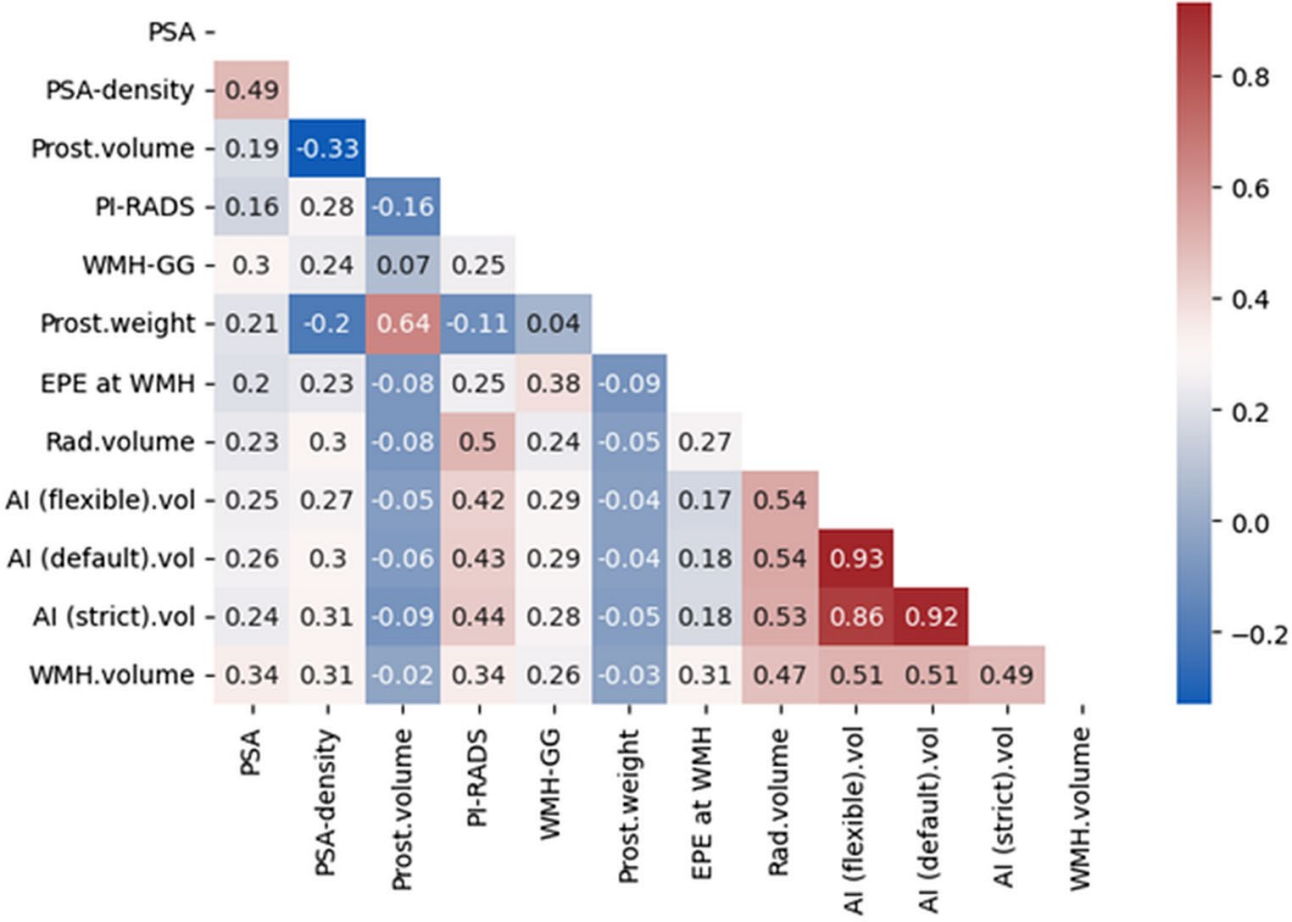
Kendall tau correlation coefficients (τ) for clinical, imaging, and pathological parameters, including volumetric measurements by radiologist and AI at flexible, default, and strict thresholds. AI: Artificial intelligence, EPE: Extraprostatic extension, GG: Grade Group, PI-RADS: Prostate Imaging Reporting and Data System, PSA: Prostate-specific antigen, WMH: Whole-mount histopathology

The PCa volume on WMH showed moderate positive correlations with radiologist volume (τ = 0.47, *P* <.001) and AI estimations at flexible (τ = 0.51, *P* <.001), default (τ = 0.51, *P* <.001), and strict (τ = 0.49, *P* <.001) thresholds. Radiologist volume also showed strong positive correlations with AI estimations at flexible (τ = 0.54, *P* <.001), default (τ = 0.54, *P* <.001), and strict (τ = 0.53, *P* <.001) thresholds. AI estimations were highly correlated with each other, with correlations of τ = 0.93 (flexible vs. default, *P* <.001), τ = 0.86 (flexible vs. strict, *P* <.001), and τ = 0.92 (default vs. strict, *P* <.001). Other factors positively associated with WMH volume included PSA (τ = 0.34, *P* <.001), PSA density (τ = 0.31, *P* <.001), ISUP GG at WMH (τ = 0.25, *P* <.001), and the presence of EPE (τ = 0.31, *P* <.001). Pairwise correlations between clinical parameters, MRI features, and volume estimation techniques are shown in Fig. 6.

## Discussion

Accurate determination of tumor volume is essential for selecting appropriate candidates for focal therapy, fine-tuning the treatment delivery, and determining the strategies for more radical treatments. An AI-based system for tumor volume estimation could complement radiologists, particularly in high-volume settings or initial assessments where rapid, reproducible estimates are valuable. By identifying cases needing closer examination, such algorithms could also allow radiologists to focus on complex cases, enhancing workflow in patient selection and treatment planning. In this study, we evaluated the performance of a bpMRI-based lesion detection & segmentation AI algorithm and a radiologist in estimating PCa volumes using whole mount histopathology as the reference standard. Our findings showed that the PCa volume on WMH (median of 2.3 mL) was underestimated by both the AI-based automated lesion segmentation model at several thresholds (range of medians, 0.7–1.2 mL; all *P* <.001) and the prospectively obtained contours by the radiologist (median of 2.1 mL, *P* =.003). While radiologist estimations demonstrated better agreement with WMH volumes than AI-based methods, significant discrepancies were still observed, particularly in patients with high-risk histopathology and imaging features. These findings not only underscore the essential role of radiologist expertise but also highlight the ongoing challenges in non-invasive tumor volume estimation, which can directly impact treatment decisions and patient outcomes.

Our findings align with a systematic review of 18 studies involving 1438 patients (27) that reported consistent underestimation of PCa volume on WMH using MRI by 17–20%. Both AI and radiologist estimations consistently underestimated disease volume in patients with elevated PSA levels, higher PI-RADS scores, higher Grade Groups, and those with extraprostatic involvement on WMH. This suggests that imaging alone may not fully capture the extent of aggressive disease, as also demonstrated by Le Nobin et al. (28), who reported more severe underestimation in patients with MRI risk scores ≥ 4 and ≥ GG2. Contrary to our findings, Pooli et al. (14) and Bratan et al. (15) suggested the extent of disease in highly suspicious MRI score lesions (i.e., PI-RADS 5, Likert 5) was better measured. However, certain methodological differences exist between these investigations and our study (14, 15). Pooli et al. (14) relied on 2D measurements, primarily in the axial plane, using only the maximum diameter of the tumor on MRI and histopathology. They also had suboptimal registration between MRI and histopathology. In contrast, our study utilized 3D measurements by both radiologists and AI, allowing a more comprehensive estimation of total tumor volume. Additionally, each patient in our study had a customized 3D-printed mold, enabling precise alignment of histopathology slides with MRI slices. Detailed histopathological mapping on digitized whole mount sections also offered a more accurate representation of the actual disease focus compared to methods used earlier such as single-dimension measurements, ellipsoid calculations, or crude ink markings (14, 16, 29). In the study by Bratan et al. (15) readers were aware that all patients had undergone radical prostatectomy and retrospectively evaluated MRI sequences individually to delineate tumor areas on each sequence. A uropathologist matched MRI slices to histopathology slides by viewing images side-by-side, and all calculations were made without accounting for tissue shrinkage. In our study, we not only accounted for tissue shrinkage but also included prospectively created radiologist contours, done prior to surgery using all mpMRI sequences. Moreover, in contrast to Sorce et al. (30), who reported underestimation of PCa volume on MRI regardless of PI-RADS scores (particularly those smaller than 2 mL), we did not observe significant discrepancies between radiologist estimations and WMH volumes for patients with PI-RADS 4 index lesions. This difference may be a result of our radiologist having access to a DCE sequence in addition to T2WI and DWI, whereas Sorce et al. used only T2WI or DWI for radiologist lesion delineation (30).

While radiologist estimations closely approximate WMH measurements in certain groups, the process is highly time-consuming. Integrating AI into the clinical workflow could enhance operational efficiency and provide consistency. However, our study reveals that the AI model tested, which previously showed promising results for lesion detection, underperformed in estimating PCa volumes compared to WMH, particularly at stricter thresholds despite displaying moderate positive correlations with WMH-based volumes (τ = 0.49–0.51, all *P* <.001). This underestimation by AI was observed across nearly all subgroups, with the exception of those with very low sample sizes, where the lack of statistical significance likely reflects insufficient power rather than comparable performance. These findings highlight the importance of transparency in recognizing AI’s limitations, as inaccuracies in critical diagnostic workups could affect treatment decisions and outcomes. Ensuring that clinicians are aware of these limitations is crucial to maintaining trust and appropriately integrating AI into clinical workflows. The AI model was designed to detect PI-RADS 2–5 lesions while maintaining a reasonable false-positive rate, rather than specifically identifying the entirety of the tumor volume as was under study in this investigation (25). This underestimation could lead to suboptimal treatment planning, underscoring the need for further refinement and validation for out-of-domain use. The observed underperformance of the AI model at stricter thresholds suggests that it is better suited as adjunct to radiologist rather than standalone solution. However, its rapid and reproducible estimates could be leveraged for initial screenings, particularly in high-volume or resource-constrained settings. Additionally, our analysis showed that clinical parameters such as PSA, PSA density, and ISUP grade group were positively correlated with WMH volumes. This hints that incorporating these factors could lead to AI models with improved accuracy in tumor volume estimation (31). Moreover, there are existing efforts in building AI tools for predicting EPE on histopathology (32, 33). Given the worse AI volume estimations in the presence of EPE, by adopting EPE-detecting algorithms from existing studies and integrating to our tumor volume estimation model to adjust for EPE involvement could potentially offer a more accurate assessment of overall tumor volume.

Another important point highlighted in our study is the identification of specific criteria for selecting patients for focal therapy (34). Our findings indicate that radiologist-derived lesion volume estimations closely matched WMH measurements in patients with PSA values less than 10 ng/mL, PI-RADS 4 lesions, gland volumes not exceeding 58 mL, and those without high-grade PCa (≥ GG4) or extraprostatic extension on WMH. These results are consistent with previous recommendations using a PSA cutoff of ≤ 10 ng/mL and ≤ GG3 for determining focal therapy eligibility (35–37). The criteria we identified could help pinpoint patients who are more likely to benefit from focal treatment, given the higher accuracy of MRI in estimating their disease volume although these patients are expected to do better anyway owing to their lower risk disease. This suggests that integrating clinical parameters and imaging features into multidisciplinary discussions or decision-support tools could further enhance the selection process for focal therapies, thereby leading to more personalized strategies and improved outcomes for this subgroup (38).

Our study has several limitations that should be considered. First, all radiology reads were prospectively conducted by a single expert genitourinary radiologist. While this ensured consistency among the reports, it may limit the generalizability of our findings as it may not reflect inter-reader variability seen in broader clinical practice. Discrepancies between radiologists and the reference standard may be even greater among less-experienced readers. Future studies involving multiple radiologists with varying levels of expertise to assess the impact of inter-reader variability on volume estimation could be insightful. Second, the sample size was restricted due to the single-center design and necessary exclusions, confining our study to patients with detailed histopathological mappings for more accurate PCa volumetric measurements. While creating such datasets demands extensive time and multidisciplinary collaboration, large datasets curated from multiple centers are still needed to validate our findings. Third, we only included patients with MRI-visible lesions as we aimed to compare the radiologist measurement with the WMH. Lastly, only one AI model was evaluated in this investigation.

In conclusion, while radiologist-derived estimations of PCa volumes generally align better with WMH compared to AI-based methods, significant discrepancies persist in both approaches. Radiologist contours can reliably estimate tumor volume in patients with PI-RADS 4 lesions and PSA values below 10 ng/mL, supporting the potential use of focal therapy. Although not yet optimal as a standalone estimator, AI could serve as a preliminary assessment tool in clinical practice, helping to triage patients for radiologist oversight. However, inconsistencies in more aggressive diseases underscore the need for continuous enhancement of AI tools. Incorporating clinical parameters, leveraging multicenter international datasets and cascading multiple AI models could enhance accuracy and advance their clinical utility across broader clinical settings.

## Electronic supplementary material

Below is the link to the electronic supplementary material.

Supplementary Material 1

## Data Availability

The dataset generated and analyzed during the current study is available from the corresponding author upon reasonable request.
