# Supplementary material for: Evaluating deep learning and radiologist performance in volumetric prostate cancer analysis with biparametric MRI and histopathologically mapped slides
Source: Abdom Radiol (NY). 2024 Dec 11;50(6):2732–44. doi: 10.1007/s00261-024-04734-6 (PMC12069428; doi:10.1007/s00261-024-04734-6)
Supplement: Supplementary file 1 — Supplementary Material 1 [file 261_2024_4734_MOESM1_ESM.docx]

**Supplemental Material**

| **Parameters** | **With ERC** | | | **Without ERC** | | |
| --- | --- | --- | --- | --- | --- | --- |
|  | **T2WI** | **High *b*-value DWI^†^** | **DCE^§^** | **T2WI** | **High *b*-value DWI^‡^** | **DCE^§^** |
| Field of view (mm) | 140 × 140 | 140 × 140 | 262 × 262 | 180 × 180 | 140 × 140 | 262 × 262 |
| Acquisition matrix | 304 × 234 | 76 × 78 | 188 × 96 | 320 × 216 | 64 × 62 | 176 × 66 |
| Repetition time (msec) | 4434 | 6987 | 3.7 | 3686 | 7218 | 3.7 |
| Echo time (msec) | 120 | 52 | 2.3 | 120 | 47 | 2.3 |
| Flip angle (degrees) | 90 | 90 | 8.5 | 90 | 90 | 8.5 |
| Section thickness (mm) | 3 | 3 | 3 | 3 | 3 | 3 |
| Image reconstruction matrix (pixels) | 512 × 512 | 256 × 256 | 256 × 256 | 512 × 512 | 256 × 256 | 256 × 256 |
| Reconstruction voxel imaging resolution (mm/pixel) | 0.27 × 0.27 × 3 | 0.55 × 0.55 × 2.73 | 1.02 × 1.02 × 3 | 0.35 × 0.35 × 3 | 1.09 × 1.09 × 3 | 1.02 × 1.02 × 3 |
| Time for acquisition (min:sec) | 2:48 | 3:50 | 5:16 | 4:48 | 6:08 | 5:16 |

**Supplemental Table - 1** Image acquisition parameters

Note: ERC = Endorectal coil, T2WI = T2-weighted imaging, DWI = Diffusion-weighted imaging, DCE = Dynamic contrast enhanced imaging

† b = 2000 sec/mm^2^

‡ b = 1500 sec/mm^2^

§ Temporal resolution is 5.6 seconds.

| Aspect | Description |
| --- | --- |
| MRI Sequences | Radiologist: T2W-MRI, DW-MRI, and DCE-MRI  AI: T2W-MRI and DW-MRI |
| Radiologist readout | Prospective readouts by an expert genitourinary radiologist |
| Measurement Approach | 3D volumetric contours for radiologist and AI; detailed mapping across multiple whole-mount slides. |
| Customized 3D Molds | Patient-specific 3D-printed molds to enable precise MRI-histopathology alignment. |
| MRI-Histopathology Registration | ProsRegNet was employed to register whole-mount slides to T2W-MRIs for accurate and reproducible alignment |
| Tissue Shrinkage Correction | A correction factor of 1.15 was applied to histopathological volumes to account for tissue shrinkage. |
| Analyses | PSA levels, PI-RADS scores, prostate volume and postsurgical features such as ISUP and EPE grade. |

**Supplemental Table 2 – Methodological Summary of Key Aspects**

AI: Artificial intelligence, DCE-MRI: Dynamic contrast-enhanced MRI, DW-MRI: Diffusion-weighted MRI, EPE: Extraprostatic extension, ISUP: International Society of Urological Pathology, PI-RADS: Prostate Imaging Reporting & Data System, PSA: Prostate-specific antigen, T2W-MRI: T2-weighted MRI

| Groups and comparisons | Median Volume (mL) | IQR | *P* value |
| --- | --- | --- | --- |
| PSA < 4 ng/mL (n=11) |  |  |  |
| WMH-based volume | 1.06 | 0.87-3.27 | Ref |
| Radiologist | 1.4 | 1.19-2.13 | .64 |
| AI (flexible) | 0.75 | 0.41-0.99 | .007 |
| AI (default) | 0.54 | 0.31-0.86 | .002 |
| AI (strict) | 0.34 | 0.26-0.72 | <.001 |
| 4 ng/mL ≤ PSA < 10 ng/mL (n=67) |  |  |  |
| WMH-based volume | 1.84 | 1.07-3.72 | Ref |
| Radiologist | 2.04 | 1.13-3.23 | .27 |
| AI (flexible) | 1.18 | 0.51-1.98 | <.001 |
| AI (default) | 0.84 | 0.37-1.48 | <.001 |
| AI (strict) | 0.65 | 0.22-1.19 | <.001 |
| 10 ng/mL ≤ PSA < 20 ng/mL (n=24) |  |  |  |
| WMH-based volume | 4.77 | 3.28-6.71 | Ref |
| Radiologist | 2.57 | 1.06-4.26 | <.001 |
| AI (flexible) | 2.05 | 0.71-3.65 | <.001 |
| AI (default) | 1.5 | 0.55-3.14 | <.001 |
| AI (strict) | 1.15 | 0.42-2.65 | <.001 |
| PSA ≥ 20 mL (n=6) |  |  |  |
| WMH-based volume | 11.62 | 6.74-15.94 | Ref |
| Radiologist | 8.07 | 6.14-12.17 | >.99 |
| AI (flexible) | 7.78 | 4.16-13.33 | .22 |
| AI (default) | 6.56 | 3.47-12.16 | .09 |
| AI (strict) | 5.57 | 2.78-10.94 | .06 |

**Supplemental Table 3 –** Comparison of prostate cancer volumes (mL) as measured by whole mount histopathology, radiologist, and AI-based estimations (flexible, default, strict thresholds) stratified by the commonly used prostate-specific antigen thresholds.

AI: Artificial intelligence, GG: ISUP grade group, PSA: Prostate-specific antigen, WMH: Whole-mount histopathology
